# Synovial fluid α-defensin in the diagnosis of periprosthetic joint infection: the lateral flow test is an effective intraoperative detection method

**DOI:** 10.1186/s13018-019-1320-9

**Published:** 2019-08-28

**Authors:** Xuequan Han, Kai Xie, Xu Jiang, Liao Wang, Haishan Wu, Xinhua Qu, Mengning Yan

**Affiliations:** 1 Shanghai Key Laboratory of Orthopaedic Implants, Department of Orthopaedic Surgery, Shanghai Ninth People’s Hospital, Shanghai Jiao Tong University School of Medicine, 639 Zhizaoju Road, Shanghai, China; 2 Department of Bone and Joint Surgery, Renji Hospital, School of Medicine, Shanghai Jiao Tong University School of Medicine, 145 Middle Shandong Road, Shanghai, China

**Keywords:** α-Defensin, Periprosthetic joint infection, Arthroplasty, Biomarker, Diagnosis

## Abstract

**Background:**

Synovial fluid α-defensin is a valuable biomarker for periprosthetic joint infection (PJI). Its diagnostic value for PJI has been widely evaluated recently, but results are inconsistent, especially for different test methods. The objective of this study was to evaluate the diagnostic value of laboratory-based immunoassay and lateral flow testing for the detection of α-defensin against hip and knee PJI.

**Methods:**

We systematically searched MEDLINE and EMBASE for articles on the diagnostic accuracy of α-defensin for PJI published up to September 2018. The pooled sensitivity, specificity, area under the curve (AUC), positive likelihood ratio (PLR), negative likelihood ratio (NLR), and diagnostic odds ratio (DOR) were calculated for the evaluation of the diagnostic value of α-defensin for PJI.

**Results:**

Nineteen studies were included. Eleven evaluated laboratory-based immunoassay, and 10 evaluated the lateral flow test results. The pooled sensitivity, specificity, AUC, PLR, NLR, and DOR of laboratory-based immunoassays were 0.96 (95% confidence interval [CI] 0.90–0.98), 0.97 (95% CI 0.95–0.99), 0.99 (95% CI 0.98–1.00), 35.0 (95% CI 18.5–66.2), 0.04 (95% CI 0.02–0.11), and 811 (95% CI 220–2990), respectively. The pooled sensitivity, specificity, AUC, PLR, NLR, and DOR of the lateral flow test were 0.86 (95% CI 0.81–0.91), 0.96 (95% CI 0.93–0.98), 0.95 (95% CI 0.93–0.97), 21.2 (95% CI 11.7–38.5), 0.14 (95% CI 0.10–0.21), and 148 (95% CI 64–343), respectively.

**Conclusions:**

Laboratory-based immunoassay of α-defensin is highly accurate for the diagnosis of hip and knee PJI. The lateral flow test is less sensitive but still a useful intraoperative detection tool for PJI.

## Background

Total joint arthroplasty (TJA) is an effective treatment for advanced joint disease [1]. However, periprosthetic joint infection (PJI) is a serious complication after TJA, which often poses a threat to patient health and leads to economic burdens [2]. Although standardized surgical procedures and perioperative management have reduced the incidence of hip and knee PJI to 1–2%, PJI is still an important reason for revision surgery [3, 4]. Previous studies reported that PJI accounted for 14.5% of revision total hip arthroplasties and 25% of revision total knee arthroplasties [5, 6]. Given the different treatment options for PJI and aseptic loosening, accurate and timely diagnosis is valuable in revision surgery [3]. Unfortunately, the identification of PJI and aseptic loosening remains a challenge due to the lack of a gold standard test. Serologic examination and bacterial culture have always been common tests for PJI [1]. However, a previous meta-analysis showed that the sensitivity and specificity of serum C-reactive protein (CRP) levels for PJI were 0.82 and 0.77, respectively [7]. In addition, the pooled sensitivity of synovial fluid aspiration culture for PJI was only 0.72 [8].

In the past few years, the diagnostic accuracy of α-defensin for PJI has been widely evaluated, and some of these results show great reliability [9–27]. Alpha-defensin is a cysteine-rich antimicrobial peptide that exists in many types of cells in the body [28–31]. As an innate immune response to the invasion of pathogens, α-defensin can be released by activated neutrophils to exert antibacterial activity [29]. One genomic study showed that the expression and release of α-defensin is a specific response of neutrophils to infectious arthritis that is not affected by non-infectious inflammation, such as acute gouty arthritis [32]. This suggests that α-defensin has the potential to be an accurate indicator in the diagnosis of PJI. Currently, synovial fluid α-defensin can be detected by both laboratory-based immunoassay and a lateral flow test. In the former, synovial fluid is sent to an advanced laboratory within 24 h and measured via standard enzyme-linked immunosorbent assay (ELISA). The lateral flow test is a rapid detection device that can be used for intraoperative PJI diagnosis.

Before this study, some meta-analyses evaluated the diagnostic value of α-defensin in PJI and showed that the laboratory-based immunoassay has a very high diagnostic value, whereas the lateral flow test is less accurate [33–38]. However, only up to six studies on the lateral flow test were included. After these studies, the diagnostic accuracy of α-defensin for PJI has been widely assessed with inconsistent results [22, 24, 26, 39–43]. Therefore, the purpose of the current meta-analysis was to reassess the diagnostic value of laboratory-based immunoassay and the lateral flow test for the detection of synovial fluid α-defensin against PJI.

## Methods

The design and implementation of this study was based on the Preferred Reporting Items for Systematic Reviews and Meta-Analyses (PRISMA) statement [44].

### Search strategy

Two independent reviewers systematically searched for articles on the diagnostic value of α-defensin in PJI in the MEDLINE and EMBASE databases from the inception of the databases until September 2018. The search terms were as follows: α-defensin, alpha-defensin, synovial fluid, biomarker, inflammatory, arthroplasty or replacement, sensitivity or specificity, septic, aseptic or aseptic loosening, prosthesis infection, infectious or infected, and diagnose or diagnostic. Additional studies were identified from the bibliographies of relevant articles.

### Eligibility criteria

Studies included in our meta-analysis complied with the following criteria: (1) studies that evaluated the accuracy of α-defensin for the diagnosis of PJI with the original or updated Musculoskeletal Infection Society (MSIS) criteria [45, 46], (2) the patients included in studies received the test clinically, (3) studies that provided data for true-positive, false-negative, false-positive, and true-negative findings for the comparison of α-defensin detection with the reference standard, (4) articles written in the English language, and (5) non-human experiments. Case reports were excluded.

The quality of all studies was independently evaluated using the Quality Assessment of Diagnostic Accuracy Studies (QUADAS) tool [47]. All divergences were negotiated with the assistance of a third investigator.

### Data extraction

Two reviewers used standardized forms to independently extract the characteristics of the included studies. The characteristics to be extracted included the following items: authors’ names, year of publication, number and mean age of patients, country in which the study was performed, study design, type of patient enrolment, test method, exclusion of patients who have been treated with antibiotics, and site of arthroplasty. A third independent reviewer helped to resolve all disagreements between the first two reviewers in the data extraction process.

### Statistical analysis

True-positive, false-negative, false-positive, and true-negative rates were extracted from the included studies. Pooled sensitivity, specificity, area under the curve (AUC), and diagnostic odds ratio (DOR) were calculated to estimate the capability to identify PJI and aseptic loosening. We used positive likelihood ratio (PLR), negative likelihood ratio (NLR), and post-test probability to assess the clinical utility of the α-defensin test for diagnosis of PJI. We calculated *I*^2^ to assess the heterogeneity of studies [48]. An *I*^2^ value > 50% indicated substantial heterogeneity among studies, and the diagnostic accuracy of α-defensin for the diagnosis of PJI was calculated using the random effects model [49]. We performed subgroup analysis to explore the influence of various factors that affect the diagnostic accuracy of the above two α-defensin tests for PJI. Deeks’ funnel plot asymmetry test was used to estimate publication bias [50]. All statistical analyses were performed using STATA version 14 (StataCorp, College Station, TX, USA).

## Results

### Search results

**Fig. 1:**
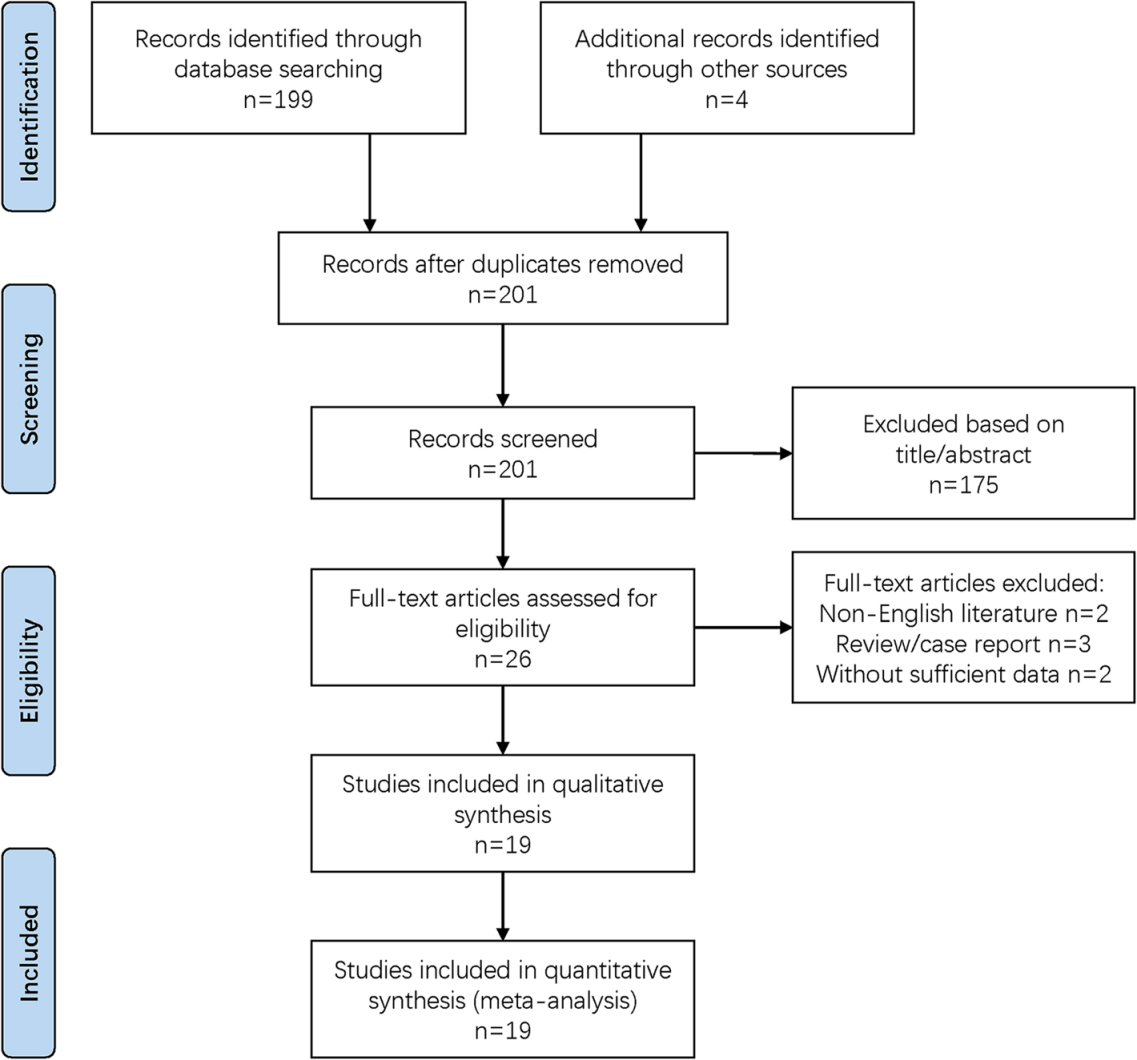
Flow diagram for study selection

We obtained 203 articles by searching databases and the bibliographies of identified articles. We excluded 175 articles after reading the title and abstract and seven articles after reading the full text (Fig. 1).

Finally, 19 articles were included in the study (Table 1). All articles were published between 2014 and 2018. These 19 studies included 2043 patients who underwent revision surgery, 609 of whom were diagnosed with PJI. Eight studies were conducted in the USA and 11 were conducted in Europe. The average age of patients in all studies ranged from 62 to 71 years. Thirteen studies were prospective designs, and the others were retrospective designs. All studies were conducted on the hip and/or knee joints and used the Musculoskeletal Infection Society (MSIS) criteria to determine the diagnosis of PJI. To detect synovial fluid α-defensin, 11 studies (1110 patients in total) used a laboratory-based immunoassay and 10 studies (933 patients in total) used the lateral flow test. In the QUADAS tool evaluation, all studies showed good quality. The characteristics of all studies are shown in Table 1.

**Table 1:** Characteristics of the 19 studies in meta-analysis for the diagnosis of PJI using α-defensin

Study	Country	Patients number	Mean age (years)	Study design	Excluded antibiotic therapy	Site of arthroplasty	Reference standard	QUADAS
Laboratory-based immunoassay								
Deirmengian et al., 2014 [10]	USA	95	67	Prospective	N	Hip, knee	MSIS (2011)	14
Bingham et al., 2014 [9]	USA	57	64.2	Retrospective	NA	Hip, knee	MSIS (2011)	13
Deirmengian et al., 2014 [10]	USA	149	65	Prospective	N	Hip, knee	MSIS (2011)	13
Deirmengian et al., 2014 [10]	USA	46	65	Prospective	N	Hip, knee	MSIS (2011)	13
Frangiamore et al., 2016 [15]	USA	78	63.3	Prospective	NA	Hip, knee	MSIS (2011)	14
Bonanzinga et al., 2017 [19]	Germany	156	NA	Prospective	N	Hip, knee	MSIS (2013)	14
Gehrke et al., 2018 [23]	Germany	173	NA	Prospective	Y	Hip, knee	MSIS (2013)	14
Kanwar et al., 2018 [24]	USA	70	66	Retrospective	NA	Hip, knee	MSIS (2013)	14
Sigmund et al., 2018 [42]	Germany	71	70	Retrospective	Y	Hip, knee	MSIS (2013)	13
Stone et al., 2018 [43]	USA	183	65.7	Retrospective	Y	Hip, knee	MSIS (2011)	14
Kelly et al., 2018 [39]	USA	32	64	Retrospective	N	Hip, knee	MSIS (2013)	13
Lateral flow test								
Kasparek et al., 2016 [16]	Austria	40	NA	Retrospective	Y	Hip, knee	MSIS (2013)	13
Suda et al., 2017 [21]	Germany	28	67.7	Prospective	N	Hip, knee	MSIS (2013)	13
Balato et al., 2018 [17]	Italy	51	63	Prospective	Y	Knee	MSIS (2013)	12
Berger et al., 2017 [18]	Belgium	121	63.5	Prospective	N	Hip, knee	MSIS (2011)	14
Gehrke et al., 2018 [23]	Germany	191	NA	Prospective	Y	Hip, knee	MSIS (2013)	14
Plate et al., 2018 [26]	Switzerland	109	65	Prospective	Y	Hip, knee	MSIS (2013)	13
de Saint Vincent et al., 2018 [22]	French	39	62	Prospective	N	Hip, knee	MSIS (2013)	12
Riccio et al., 2018 [41]	Italy	71	69	Retrospective	N	Hip, knee	MSIS (2013)	13
Sigmund et al., 2018	Germany	71	70	Retrospective	Y	Hip, knee	MSIS (2013)	13
Renz et al., 2018 [40]	Germany	221	70	Prospective	N	Hip, knee	MSIS (2013)	14

*PJI* periprosthetic joint infection, *NA* not available, *MSIS* Musculoskeletal Infection Society, *QUADAS* Quality Assessment of Diagnostic Accuracy Studies

### Diagnostic accuracy of α-defensin for PJI

**Fig. 2:**
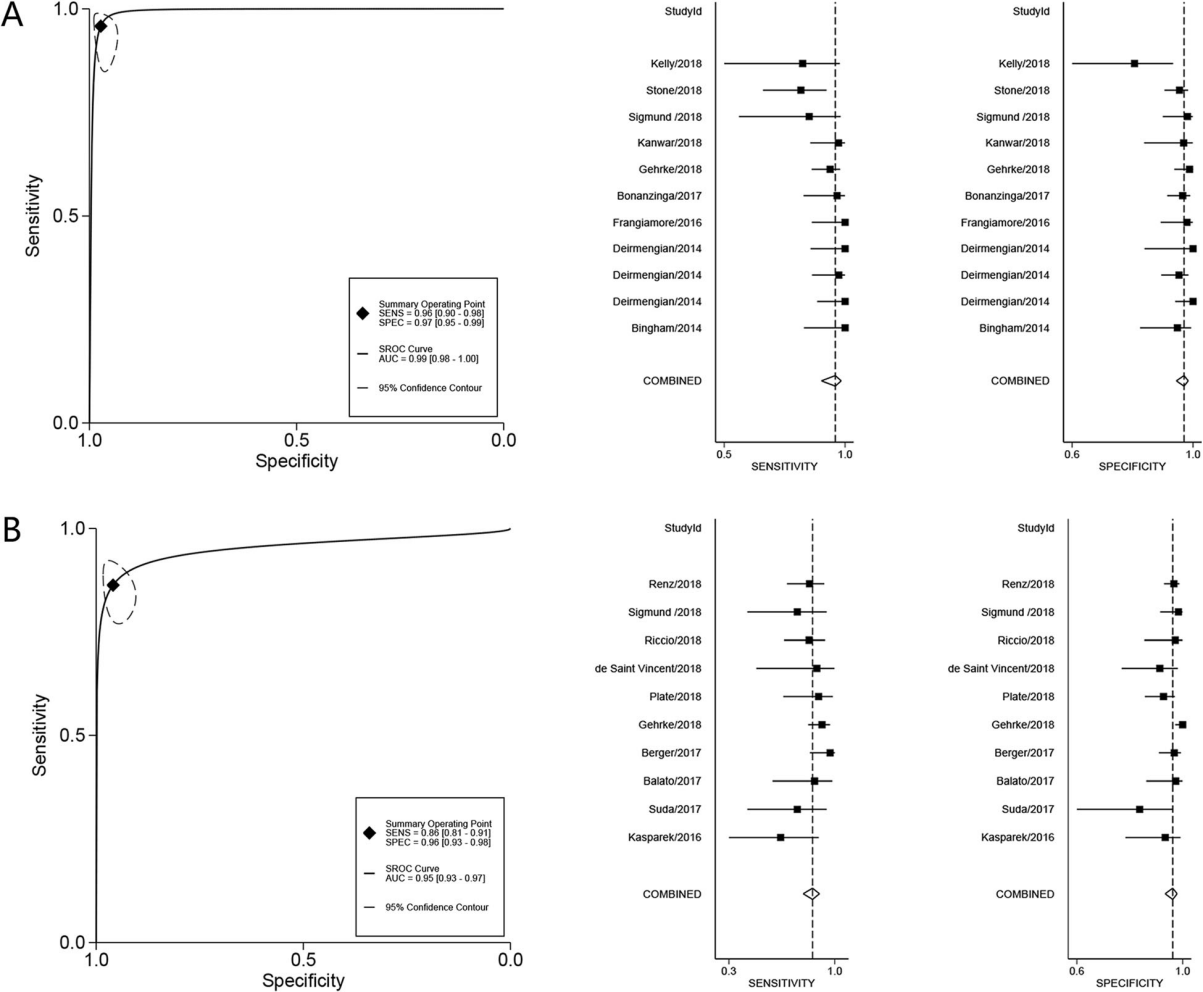
Summary receiver-operating characteristic curves and forest plots for laboratory-based immunoassay (**a**) and lateral flow test (**b**)

For the laboratory-based immunoassay, the pooled diagnostic sensitivity and specificity for PJI were 0.96 (95% confidence interval [CI] 0.90–0.98) and 0.97 (95% CI 0.95–0.99), respectively. The pooled DOR and AUC were 811 (95% CI 220–2990) and 0.99 (95% CI 0.98–1.00), respectively (Fig. 2). For the lateral flow test, the pooled sensitivity and specificity were 0.86 (95% CI 0.81–0.91) and 0.96 (95% CI 0.93–0.98), respectively. The pooled DOR and AUC were 148 (95% CI 64–343) and 0.95 (95% CI 0.93–0.97), respectively (Fig. 2). The *I*^2^ values for the laboratory-based immunoassay and lateral flow test were both 0%, indicating no potential heterogeneity.

### Clinical utility of α-defensin for PJI

**Fig. 3:**
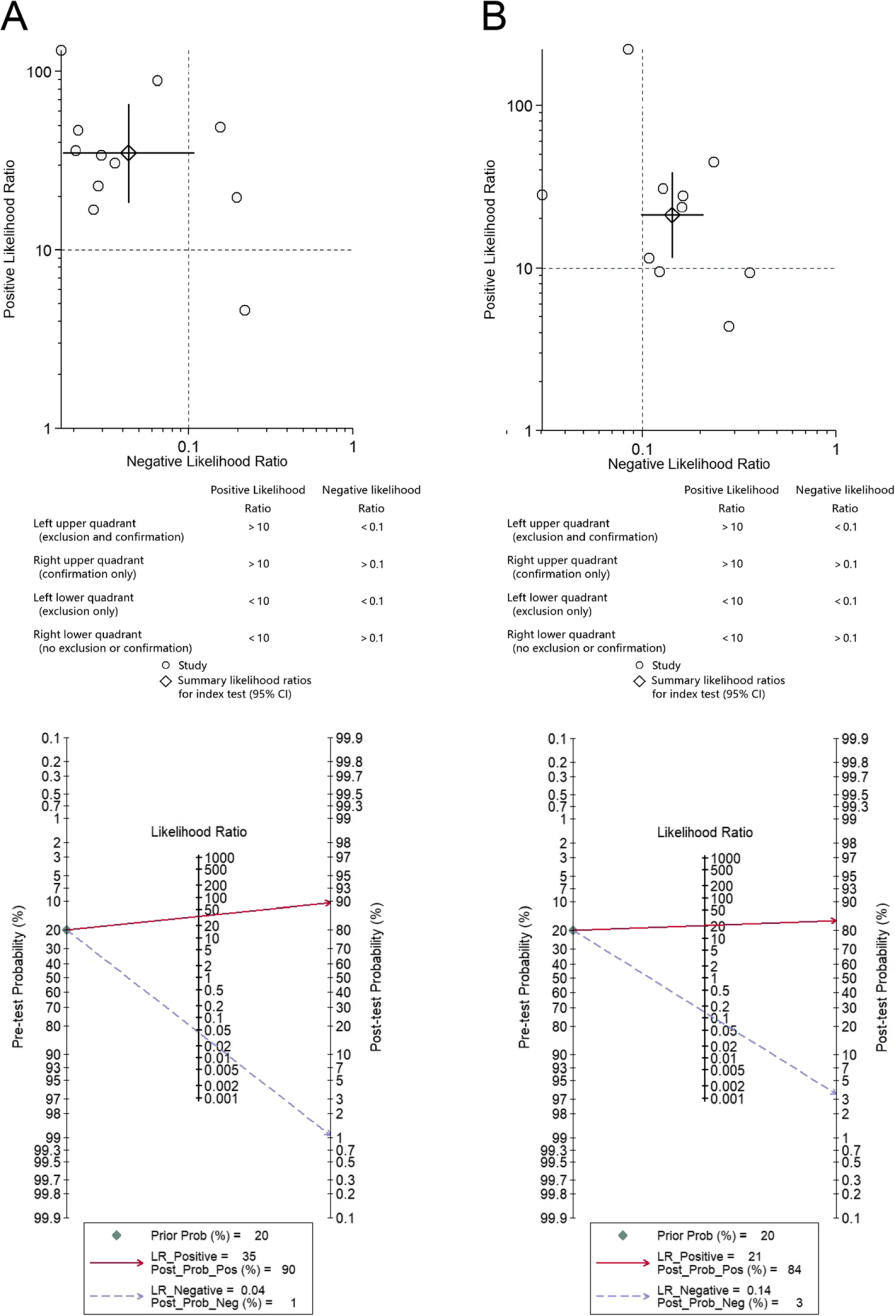
Likelihood ratio scatter diagrams and post-test probabilities for laboratory-based immunoassay (**a**) and lateral flow test (**b**)

For the laboratory-based immunoassay, the pooled PLR and NLR were 35 (95% CI 18.5–66.2) and 0.04 (95% CI 0.02–0.11), respectively. Based on the assumption that the pre-test probability was 20%, the post-test probability of PJI was 90% and 1% for the laboratory-based immunoassay, indicating positive and negative test results, respectively (Fig. 3). For the lateral flow test, the pooled PLR and NLR were 21.2 (95% CI 11.7–38.5) and 0.14 (95% CI 0.10–0.21), respectively. When the lateral flow test showed positive and negative test results, the post-test probability of PJI was 84% and 3%, respectively (Fig. 3).

### Subgroup analysis

All results from the subgroup analysis are shown in Table 2. For laboratory-based immunoassay, the diagnostic accuracy of α-defensin for PJI in the studies that excluded metallosis was higher than that in the studies that included patients with metallosis. The sensitivity and specificity of the former group were 0.97 (95% CI 0.88–0.99) and 0.99 (95% CI 0.96–1.00), respectively. The sensitivity and specificity of the latter group were 0.94 (95% CI 0.84–0.98) and 0.96 (95% CI 0.94–0.97), respectively. In addition, the diagnostic accuracy of immunoassay for the diagnosis of PJI was higher in prospective studies compared to retrospective studies. The pooled sensitivity and specificity of the prospective studies were 0.97 (95% CI 0.92–0.99) and 0.98 (95% CI 0.96–0.99), respectively. The pooled sensitivity and specificity of the retrospective studies were 0.91 (95% CI 0.79–0.96) and 0.95 (95% CI 0.90–0.98), respectively.

**Table 2:** Subgroup analysis of laboratory-based immunoassay and lateral flow test for PJI diagnosis

Subgroup analyses	No. of studies	No. of patients	Sensitivity (95%CI)	Specificity (95%CI)	AUC (95%CI)	PLR (95%CI)	NLR (95%CI)	DOR (95%CI)
Laboratory-based immunoassay								
Overall studies	11	1110	0.96 (0.90–0.98)	0.97 (0.95–0.99)	0.99 (0.98–1.00)	35.0 (18.5–66.2)	0.04 (0.02–0.11)	811 (220–2990)
Excluded metallosis								
Yes	4	416	0.97 (0.88–0.99)	0.99 (0.96–1.00)	0.99 (0.98–1.00)	80.7 (26.0–251.1)	0.03 (0.01–0.13)	2447 (383–15,647)
No and NA	7	694	0.94 (0.84–0.98)	0.96 (0.94–0.97)	0.98 (0.96–0.99)	23.1 (14.2–37.6)	0.06 (0.02–0.17)	382 (103–1414)
Study design								
Prospective	6	697	0.97 (0.92–0.99)	0.98 (0.96–0.99)	0.99 (0.98–1.00)	42.9 (22.9–80.4)	0.03 (0.01–0.09)	1480 (423–5172)
Retrospective	5	413	0.91 (0.79–0.96)	0.95 (0.90–0.98)	0.98 (0.96–0.99)	19.9 (8.9–44.5)	0.10 (0.04–0.24)	207 (52–830)
Lateral flow test								
Overall studies	10	933	0.86 (0.81–0.91)	0.96 (0.93–0.98)	0.95 (0.93–0.97)	21.2 (11.7–38.5)	0.14 (0.10–0.21)	148 (64–343)
Excluded antibiotic therapy								
Yes	5	462	0.86 (0.77–0.92)	0.97 (0.91–0.99)	0.94 (0.92–0.96)	32.7 (9.3–114.6)	0.15 (0.08–0.25)	225 (46–1099)
No and NA	5	471	0.87 (0.78–0.92)	0.95 (0.91–0.97)	0.97 (0.95–0.98)	17.3 (9.1–33.1)	0.14 (0.08–0.23)	124 (46–336)
Number of patients								
≥ 50	7	826	0.89 (0.84–0.92)	0.97 (0.94–0.99)	0.95 (0.92–0.96)	30.9 (15.4–61.9)	0.12 (0.08–0.17)	263 (109–631)

*PLR* positive likelihood ratio, *NLR* negative likelihood ratio, *DOR* diagnostic odds ratio, *AUC* area under the curve

For the lateral flow test, the diagnostic accuracy of α-defensin for PJI was similar in studies that excluded patients receiving antibiotic therapy and studies that included patients treated with antibiotics. The sensitivity and specificity of the former group were 0.86 (95% CI 0.77–0.92) and 0.97 (95% CI 0.91–0.99), respectively. The sensitivity and specificity of the latter group were 0.87 (95% CI 0.78–0.92) and 0.95 (95% CI 0.91–0.97), respectively.

### Publication bias

**Fig. 4:**
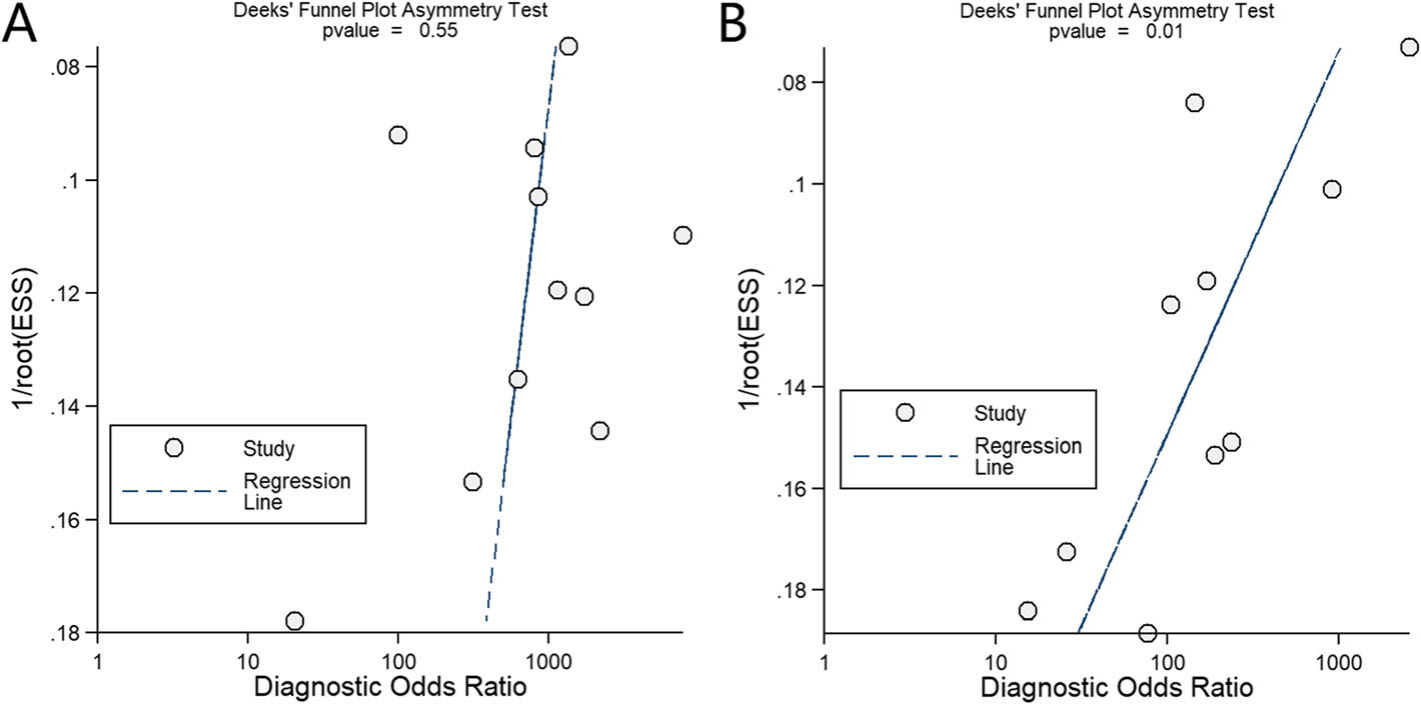
Funnel plots for the included studies: laboratory-based immunoassay (**a**) and lateral flow test (**b**)

There were potential publication biases in the studies of lateral flow test (*p* = 0.01) and no laboratory-based immunoassay (*p* = 0.55) (Fig. 4).

## Discussion

The current meta-analysis showed that synovial fluid α-defensin is a valuable indicator for hip and knee PJI. Laboratory-based immunoassay can provide a reliable preoperative diagnostic basis for the presence or absence of PJI due to its extremely high sensitivity (0.96) and specificity (0.97). Despite the low sensitivity (0.86) of the lateral flow test, it is still a good intraoperative confirmation tool for PJI based on its excellent specificity (0.96).

Accurate and timely diagnosis of PJI can avoid delays in PJI treatment on the one hand, and unnecessary surgical trauma and economic losses on the other. Unfortunately, traditional methods are often difficult to distinguish PJI from aseptic loosening. The current meta-analysis showed that laboratory-based immunoassay for the detection of α-defensin has very high diagnostic accuracy for PJI, with a sensitivity and specificity of 0.96 and 0.97, respectively. To the best of our knowledge, no method has been reported to have such a high diagnostic accuracy for PJI (Table 3). In addition, a previous study demonstrated that the gene expression of α-defensin in neutrophils is a specific immune response to infectious inflammation, which is not affected by non-infectious inflammation and the use of antibiotics [32]. Subsequent diagnostic studies have confirmed this observation [10–12, 19, 51]. Furthermore, a large-sample (1937 samples) study conducted by Deirmengian et al. [13] showed that the test has consistent diagnostic accuracy for PJI regardless of the organism type, gram type, species, or virulence of the organism. However, the only study for shoulder PJI showed that the laboratory-based immunoassay has low sensitivity (0.63) [14]. The authors of that study believed that the more commonly indolent organisms in shoulder PJI were responsible for its low sensitivity, but this conjecture was inconsistent with previous studies. The diagnostic accuracy of laboratory-based immunoassay for shoulder PJI requires additional studies. In addition, this test requires the delivery of a synovial fluid sample to an advanced laboratory for standard ELISA within 24 h. Therefore, the time delay and economic costs need to be considered before performing this test.

**Table 3:** Diagnostic value of different diagnostic method for the diagnosis of PJI

Diagnostic method	Study	Sensitivity (95% CI)	Specificity (95% CI)	PLR (95% CI)	NLR (95% CI)	DOR (95% CI)	AUC (95% CI)
Synovial fluid biomarkers and aspiration culture							
Aspiration culture	Qu et al., 2013 [8]	0.72 (0.65–0.78)	0.95 (0.93–0.97)	15.3 (10.6–22.1)	0.29 (0.23–0.38)	52.00 (31.00–86.00)	0.94 (0.92–0.96)
White cell count	Qu et al., 2014 [52]	0.88 (0.81–0.93)	0.93 (0.88–0.96)	13.30 (7.70–22.80)	0.13 (0.08–0.21)	103.00 (54.00–197.00)	0.96 (0.94–0.98)
Polymorphonuclear	Qu et al., 2014 [52]	0.90 (0.84–0.93)	0.88 (0.83–0.92)	7.60 (4.90–11.70)	0.12 (0.07–0.19)	64.00 (27.00–149.00)	0.95 (0.93–0.96)
C-reactive protein	Wang et al., 2016 [53]	0.92 (0.86–0.96)	0.90 (0.87–0.93)	9.00 (6.15–13.16)	0.10 (0.06–0.18)	101.40 (48.07–213.93)	0.97 NA
Leukocyte esterase	Wyatt et al., 2016 [34]	0.81 (0.49–0.95)	0.97 (0.82–0.99)	23.90 (3.80–152.10)	0.19 (0.06–0.66)	NA	0.97 (0.95–0.98)
Interleukin-6	Xie et al., 2017 [54]	0.91 (0.82–0.96)	0.90 (0.84–0.95)	9.50 (5.40–17.20)	0.09 (0.04–0.21)	101.00 (28.00–358.00)	0.96 (0.94–0.98)
Alpha-defensin (Immunoassay)	Current study	0.96 (0.90–0.98)	0.97 (0.95–0.99)	35.0 (18.5–66.2)	0.04 (0.02–0.11)	811 (220–2290)	0.99 (0.98–1.00)
Alpha-defensin (lateral flow test)	Current study	0.86 (0.81–0.91)	0.96 (0.93–0.98)	21.2 (11.7–38.5)	0.14 (0.10–0.21)	148 (64–343)	0.95 (0.93–0.97)
Serum biomarkers							
White cell count	Berbari et al., 2010 [55]	0.45 (0.41–0.49)	0.87 (0.85–0.89)	NA	NA	4.40 (2.90–6.60)	NA
Erythrocyte sedimentation rate	Berbari et al., 2010 [55]	0.75 (0.72–0.77)	0.70 (0.68–0.72)	NA	NA	7.20 (4.70–10.90)	NA
C-reactive protein	Yuan et al., 2014 [56]	0.82 (0.80–0.84)	0.77 (0.76–0.78)	3.66 (2.92–4.59)	0.26 (0.20–0.33)	17.01 (11.38–25.44)	0.88 (0.86–0.89)
Procalcitonin	Xie et al., 2017 [33]	0.53 (0.24–0.80)	0.92 (0.45–0.99)	6.80 (1.00–48.10)	0.51 (0.31–0.84)	13.00 (3.00–70.00)	0.76 (0.72–0.80)
Interleukin-6	Xie et al., 2017 [54]	0.72 (0.63–0.80)	0.89 (0.77–0.95)	6.40 (2.90–14.10)	0.31 (0.22–0.44)	20.00 (7.00–58.00)	0.83 (0.79–0.86)
Nuclear medicine							
Bone scintigraphy	Ouyang et al., 2014 [57]	0.83 (0.72–0.90)	0.73 (0.65–0.80)	3.10 (2.40–4.10)	0.23 (0.14–0.38)	14.00 (7.00–26.00)	0.85 (0.81–0.87)
Anti-granulocyte scintigraphy	Xing et al., 2013 [58]	0.83 (0.79–0.87)	0.79 (0.75–0.83)	3.56 (2.42–5.23)	0.26 (0.19–0.37)	18.76 (10.45–33.68)	0.88 NA
Leukocyte scintigraphy	Verbern et al., 2016 [59]	0.88 NA	0.92 NA	NA	NA	NA	NA
FDG-PET	Verbern et al., 2016 [59]	0.86 NA	0.93 NA	NA	NA	NA	NA
Other tests with biopsy							
Frozen section histopathology	Tsaras et al., 2012 [51]	NA	NA	12.00 (8.40–17.20)	0.23 (0.15–0.35)	54.7 (31.2–95.7)	NA
PCR assays	Qu et al., 2014 [52]	0.86 (0.77–0.92)	0.91 (0.81–0.96)	9.10 (4.60–18.20)	0.16 (0.10–0.25)	59.00 (29.00–118.00)	0.94 (0.91–0.95)
Sonication fluid cultures	Zhai et al., 2014 [60]	0.80 (0.74–0.84)	0.95 (0.90–0.98)	17.20 (7.60–38.70)	0.21 (0.17–0.27)	81.00 (35.00–186.00)	0.89 (0.86–0.91)
Gram staining	Ouyang et al., 2015 [61]	0.19 (0.12–0.27)	1.00 (0.99–1.00)	41.60 (15.50–111.20)	0.82 (0.75–0.89)	51.00 (18.00–140.00)	0.89 (0.86–0.91)

If one diagnostic method was reported by more than one meta-analysis, the most detailed and/or recent one was included in this table. *PLR* positive likelihood ratio, *NLR* negative likelihood ratio, *DOR* diagnostic odds ratio, *AUC* area under the curve, *Immunoassay* laboratory-based immunoassay, *FDG-PET* 18F-fluoro-2-deoxyglucose positron emission tomography, *PCR* polymerase chain reaction

Recently, a new method of lateral flow testing for the detection of synovial fluid α-defensin has become available. This test is easy to use and provides results after just 10 min [23]. Therefore, this test could compensate for the shortcomings of laboratory-based immunoassay (time delay) and could therefore be used for the intraoperative diagnosis of PJI. One previous meta-analysis (three studies included) showed low diagnostic efficiency, with the sensitivity and specificity of this test being 77% and 91% [36]. The current study (nine studies included) showed a more promising result with the sensitivity and specificity of 86% and 96%, respectively. Notably, the earliest three studies reported low sensitivity (67–77%) for the lateral flow test to detect PJI [16, 20, 21]. One of the studies [20] involved 15 patients (a total of 49 patients in the entire study) with a spacer in the studied joint, which may be a possible reason for the low sensitivity of the test. Based on the specificity of 0.96, the lateral flow test is a valuable intraoperative confirmation tool for PJI. However, the cost-effectiveness of this test must be considered because of its high price [34, 36, 62].

It is worth noting that several studies included in the current meta-analysis showed that the presence of a communicating sinus tract increases the false-negative rate of the α-defensin test for PJI [19, 23, 40, 42, 43]. The reason for this may be that continuous drainage reduces the concentration of α-defensin in the synovial fluid. However, a communicating sinus is one of the major MSIS criteria and results in the diagnosis of PJI. Thus, the appearance of a communicating sinus will not have a negative impact on the application of α-defensin testing in clinical practice. In contrast, this phenomenon indicates that α-defensin testing has potentially higher diagnostic efficacy in clinical practice than the summary results of the current study.

In addition, regardless of which method of α-defensin testing is used, it is necessary to guard against the presence of metallosis (adverse local tissue reaction) and crystal deposition diseases. Several studies have demonstrated that the presence of metallosis can greatly increase the likelihood of a false-positive α-defensin result [11, 16, 19, 25, 43]. Deirmengian et al. [11] proposed the simultaneous detection of synovial fluid CRP levels to correct false-positive α-defensin results. In addition, there have been reports of a tendency for false-positive α-defensin detection in cases involving crystal deposition joint diseases, such as calcium pyrophosphate dihydrate crystal deposition disease [26]. Therefore, the accurate diagnosis of periprosthetic infections still depends on thorough examination and evaluation by orthopedists.

The current study had some limitations. First, most studies do not have long-term follow-ups for potential infection cases, which possibly increased the rate of false-negatives. Second, the included studies contained patients with acute and/or chronic PJI, whose joint fluid defensin levels may differ. The diagnostic value of these two test methods for acute and chronic PJI needs to be evaluated by specially designed studies in the future. Finally, there was potential publication bias in studies on both test methods, and this may have reduced the credibility of the findings of this study.

## Conclusions

The current meta-analysis indicated that the laboratory-based immunoassay of synovial fluid α-defensin has extremely high diagnostic accuracy for hip and knee PJI. This method can improve the diagnostic ability of orthopedists when attempting to distinguish between PJI and aseptic loosening before revision surgery. The lateral flow test can be used as a confirmatory test for the intraoperative detection of PJI due to its excellent specificity. However, the cost-effectiveness of these two tests needs to be considered before use.

## Data Availability

The data in this study can be obtained free of charge using the search strategy in the “Methods” section.

## Abbreviations

AUC: Area under the curve
CRP: C-reactive protein
DOR: Diagnostic odds ratio
ELISA: Enzyme-linked immunosorbent assay
FDG-PET: 18F-Fluoro-2-deoxyglucose positron emission tomography
MSIS: Musculoskeletal Infection Society
NA: Not available
NLR: Negative likelihood ratio
PCR: Polymerase chain reaction
PJI: Periprosthetic joint infections
PLR: Positive likelihood ratio
PRISMA: Preferred Reporting Items for Systematic Reviews and Meta-Analyses
QUADAS: Quality Assessment of Diagnostic Accuracy Studies
